# Effect of Ondansetron on the Occurrence of Hypotension and on Neonatal Parameters during Spinal Anesthesia for Elective Caesarean Section: A Prospective, Randomized, Controlled, Double-Blind Study

**DOI:** 10.1155/2015/158061

**Published:** 2015-01-08

**Authors:** Walid Trabelsi, Chihebeddine Romdhani, Haythem Elaskri, Walid Sammoud, Mohamed Bensalah, Iheb Labbene, Mustapha Ferjani

**Affiliations:** Department of Anesthesia and Intensive Care Unit, Tunisian Military Hospital, 1008 Tunis, Tunisia

## Abstract

To prevent hypotension during spinal anesthesia for caesarean section, we assessed IV ondansetron of invasive maternal hemodynamic and fetal gazometric parameters.

## 1. Introduction

Spinal anesthesia has become the gold standard anesthetic technique for elective caesarean section. It is a simple, fast performed, powerful, and reliable technique. The main problem is hypotension associated with a decrease in cardiac output and uteroplacental flow which may induce fetal morbidity [1]. It is therefore crucial to prevent and/or to treat it quickly and effectively.

The spinal anesthesia for cesarean indeed requires a sensory block until T5, which always leads to an extended sympathetic block and hypotension occurs in 55% to 90% of cases, despite the partial left lateral decubitus (with the objective of limiting the aortocaval compression caused by the gravid uterus).

The main treatment is the vascular filling with crystalloid or starches and use of vasopressors. However, many studies [2–5] showed that it was inefficient and a recent review found that no intervention reliably prevents hypotension during spinal anesthesia for caesarean section [6].

The physiopathological mechanism involved in the occurrence of hypotension is systemic vascular resistance and central venous pressure from sympathetic block with vasodilation [7–11]. Bradycardia can occur from shift in cardiac autonomic balance toward the parasympathetic system, from activation of left ventricular mechanoreceptors from a sudden decrease in left ventricular volume (Bezold-Jarisch reflex) (BJR).

Pharmacological and animal studies [12] suggest that 5-HT (serotonin) may be an important factor associated with inducing the BJR and this effect can be blocked at the 5-HT3 receptor [13].

Based on these considerations, this randomized, controlled, double-blind study was performed to investigate the use of intravenous ondansetron for prophylaxis of hypotension after spinal anesthesia in parturients scheduled for elective caesarean section and its consequences on newborns' parameters.

## 2. Material and Methods

After local ethics committee approval (provided by Tunisian Military Hospital Ethics Committee, Tunis, Tunisia), recording in the Australian New Zealand Clinical Trials Registry with an assigned number of ACTRN12613000036718, and written informed consent, ASA physical status class I primipare parturients undergoing elective caesarean section at term were enrolled in this prospective, randomized, controlled, double-blinded study.

Exclusion criteria were emesis gravidarum, contraindication to spinal anesthesia (patient refusal, unstable hemodynamic, and coagulation abnormalities), chronic hypertension or preeclampsia, morbid obesity, and/or any study drugs allergy.

Patients were randomized (during preanesthetic consultation) to one of 2 groups using a random sequence (https://www.random.org/): group O received intravenously 4 mg ondansetron in 10 mL saline, 5 minutes before spinal puncture, and the control group received 10 mL of saline in the same way and same timing.

On arrival to the operating room, standard monitoring was applied and an arterial catheter was placed into the radial artery after infiltration of lidocaine (10–20 mg). Baseline (*t*_0_) measurements of noncalibrated invasive SAP, DAP, MAP, and HR were recorded.

Stroke volume (SV), cardiac output (CO), systemic vascular resistance (SVR), and other estimated variables based on continuous arterial waveform analysis system (*dP*/*dt* max, cardiac cycle efficiency (CCE) and pulse pressure variation (PPV)) were recorded, using MOSTCARE (MOSTCARE; Vytech Health, France). This new low-invasive method is capable of deriving CO from the analysis of arterial pulse wave (pulse contour method (PCM)) and allows beat-by-beat hemodynamic parameters' determination using an uncalibrated PCM named pressure recording analytical method (PRAM).

Then, a fast infusion of 10 mL/kg of saline solution was completed approximately 5 minutes before anesthesia.

The anesthesiologist in charge of the patient and performing anesthesia was blind to patient's group like all other involved personnel except for the nurse preparing the syringe and injecting the solution according to random table made using Internet-free LOGICIEL (https://www.random.org/).

With patients in the sitting position, spinal anesthesia was performed at the L2-L3 or L3-L4 with a 25 G Whitacre needle (VYGON, France), and, 5 minutes after the administration of ondansetron/placebo solution, patients received 2 mL of a hyperbaric 5 mg/mL bupivacaine solution (Bupivacaine, UNIMED Tunisie) and 0.5 mL of a 5 *μ*g/mL sufentanil solution.

Parturients were placed in a supine position immediately after the injections were completed. A left lateral tilt was applied by default to all parturients and a sensory block was assessed according to loss of pinprick sensation (25 G hypodermic needle) every 5 minutes for 15 minutes, beyond which parturients were excluded if sensory level was below T6. Surgery started as soon as the T6 dermatome was anesthetized; patients who failed to reach at least this level were excluded from the study, and general anesthesia was then administered at the attending anesthesiologist's discretion.

All hemodynamic parameters were recorded every 2 minutes to 20 minutes and then every 5 minutes until skin closure.

Hypotension, defined as a decrease from baseline values of ≥20% in systolic arterial pressure or SAP < 80 mmHg, was treated by an infusion of crystalloids (100 mL) and ephedrine bolus (6 mg) until restoration of baseline values. Ephedrine was chosen because of local unavailability of phenylephrine.

Bradycardia, defined as a 30% drop in HR or ≤45 bpm, was also treated with fluids and ephedrine up to 25 mg; if bradycardia did not resolve within 30 seconds of treatment, IV atropine 0.5 mg was given every 30 seconds until resolution. We took note of total dose of atropine and ephedrine needed.

Clinical manifestations (retching or vomiting) and/or any requests for antiemetic medication (until end of surgery) were considered as episodes of nausea-vomiting.

An investigator who was not involved with intraoperative care performed a blood gas analysis of the umbilical artery immediately after clamping of the umbilical fetal blood lactate and pH. Apgar score was at 1, 3, 5, and 10 minutes (as recommended in our institution) and newborn weight was also noted.

After delivery, patients were advised to request analgesics as soon as significant pain developed in the operated area, with a subjective numerical rating scale (NRS) score > 3 out of 10. They were prescribed IV paracetamol 1000 mg four times daily starting from their report of pain; nefopam 20 mg intravenously was then available on request every 6 h and was infused slowly over 20 minutes.

## 3. Sample Size Calculation and Statistical Analysis 

Sahoo et al. [14] found that the MAP in placebo group was 82.2 ± 10.5 mmHg. With an alpha error of 5% and power of 80% and assuming a 10% decrease in the MAP, we estimated that 26 patients would be needed in each group. Therefore 80 patients were included (40 patients in each group).

*Statistical Analysis.* We compared categorical data, between the groups, using Pearson's *χ* ^2^ test. Categorical data was described by count (percentages). We evaluated the data distribution using the Kolmogorov-Smirnov test. We used one-way analysis of variance for normally distributed continuous variables. We compared groups using Kruskall-Wallis test for nonnormally distributed continuous variables (pairwise comparison was performed by Mann-Whitney *U* test). Continuous variables were expressed as mean and standard deviation (SD) or median and interquartile range, depending on the normality distribution of the data. A *P* value of less than 0.05 was considered significant. All statistics were done using R v.2.15.1 (R Development Core Team 2012).

## 4. Results

**Figure 1:**
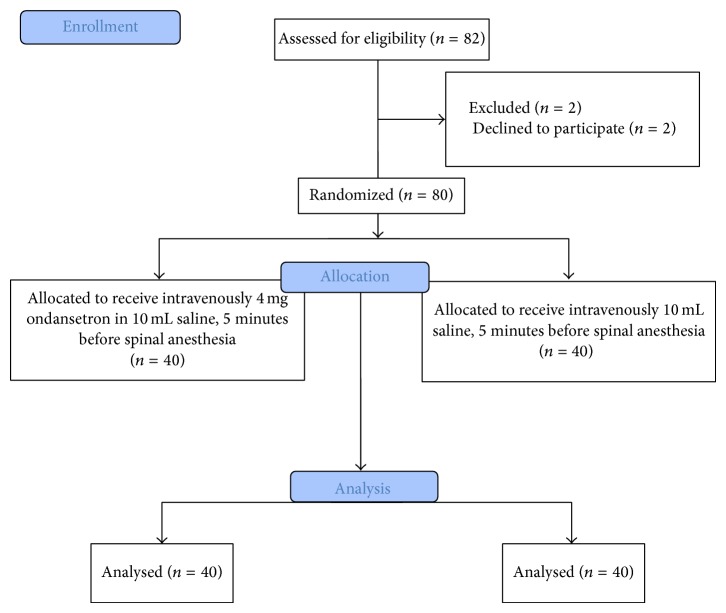
Flowchart of parturients enrolled in the study.

The flowchart of patients consented and recruited is shown in Figure 1. There were no significant differences between groups in terms of parturients physical characteristics and newborns' weight (Table 1).

**Figure 2:**
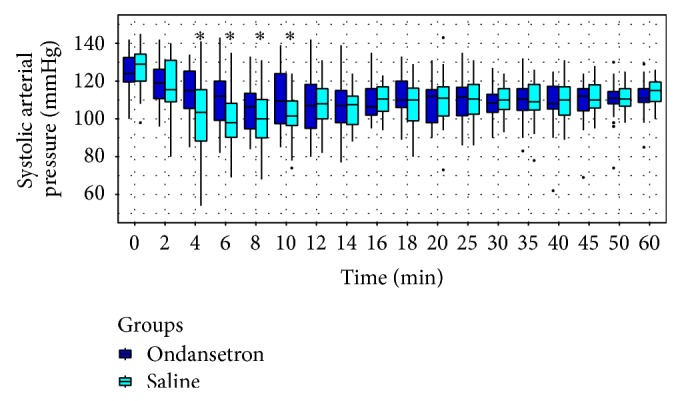
Trends of systolic blood pressure (SAP). ∗ < 0.05.

**Figure 3:**
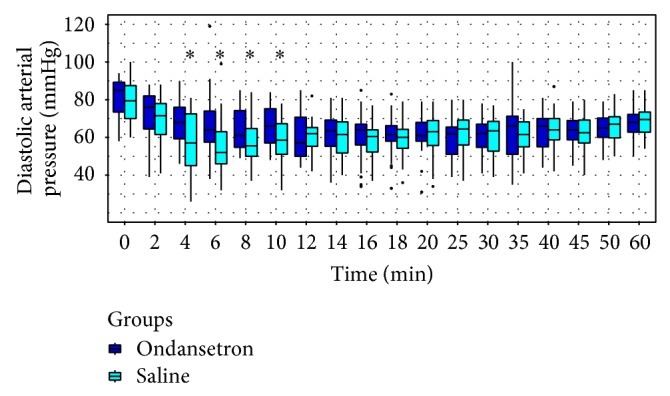
Trends of diastolic blood pressure (DAP). ∗ < 0.05.

**Figure 4:**
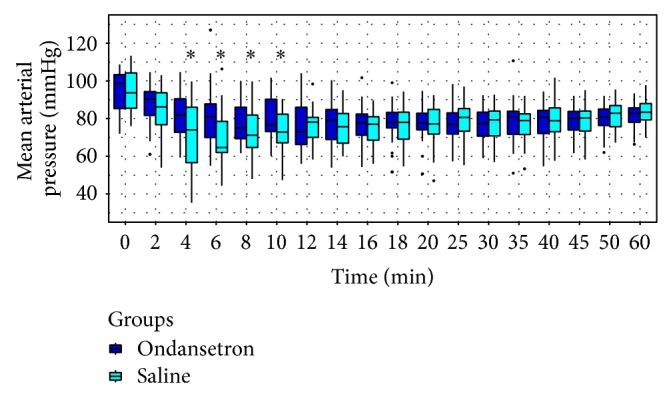
Trends of mean blood pressure (MAP). ∗ < 0.05.

SAP, DAP, and MAP were higher in group O than in group S between the 4th and 10th minutes and no difference was found until the 60th minute (Figures 2, 3, and 4, resp.).

Fewer patients in the O group experienced hypotension as compared to those in the S group: 15 (37.5%) and 31 (77.5%) (*P* < 0.001). Thus, the average consumption of ephedrine intraoperatively was 5.10 ± 7.78 mg in group O while it was 12.90 ± 9.24 mg in group S with a significant difference (*P* < 0001).

HRs were similar in both groups and bradycardia was observed in 6 patients in group O (15%), whereas it was more frequent in the S group (15 cases, 37.5%) with a significant difference (*P* = 0.022). Atropine consumption of in group S was 0.12 ± 0.22 mg. Yet, no atropine was required in group O.

Hemodynamic parameters recorded with MOSTCARE were as below. *dP*/*dt* MAX in group O was significantly higher (*P* < 0.001) than that in group S only until the 12th minute (Figure 6(a)). In addition, Cce was stable in group O while it decreased up to 40% from baseline to the 10th minute in group S (Figure 6(b)).

Both groups showed comparable small decrease in the CO not exceeding 28% between the second and the 12th minutes (Figure 6(c)). On the other hand, parturients in group S showed a major decrease in SV from baseline to the 12th minute (not observed in group O) (Figure 6(d)).

PPV was more pronounced in group S between the third and the 12th minutes (peak variation of 22% at the 6th minute). This variation did not exceed 9% in group O (Figure 6(e)).

Concerning afterload, SVR decreased more significantly in group S from the 5th minute to the 8th minute, and then no differences were noted between both groups (Figure 6(f)).

Fewer patients in group O experienced nausea and vomiting as compared to those in group S: 9 (22.5%) and 25 (62.5%), respectively (*P* < 0.001).

Apgar scores in group O were higher than those in group S until the fifth minute after birth (Figure 7). In addition, lactate of newborns whose mothers belonged to group O was lower than newborns whose mothers belonged to group S (2.09 ± 0.552 versus 3.22 ± 1.158, resp.; *P* < 0.001). Also noted is that pH of blood from the umbilical artery was closer to physiologic ranges in group O than in group S (7.38 ± 0.045 versus 7.35 ± 0.047, resp.; *P* = 0.01).

## 5. Discussion

**Figure 5:**
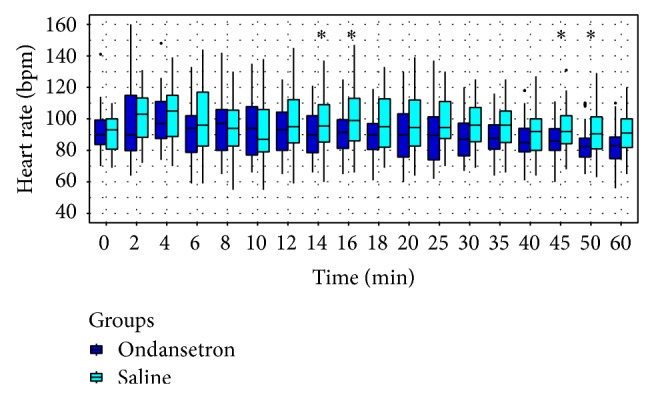
Trends of heart rate (HR). ∗ < 0.05.

**Figure 6:**
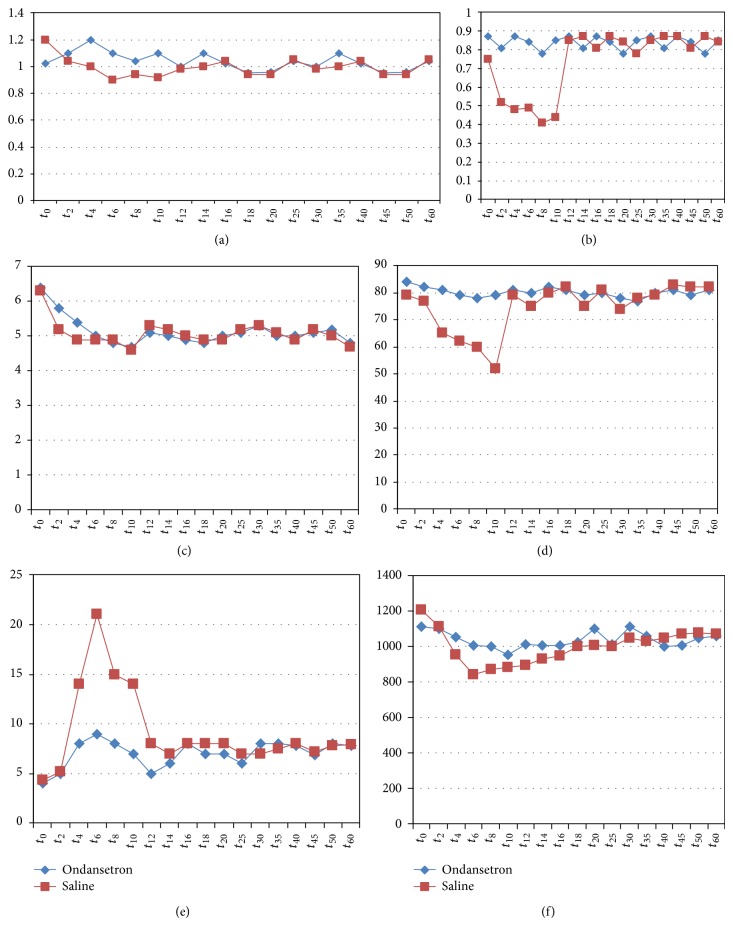
(a) Trends of mean *dP*/*dt* MAX. (b) Trends of mean cardiac cycle efficiency (CCE). (c) Trends of cardiac output (L·min^−1^). (d) Trends of mean stroke volume (mL·min^−1^). (e) Trends of mean pulse pressure variation. (f) Trends of mean systemic vascular resistance (dynes·s·cm^−5^). *t*: time in minutes.

**Figure 7:**
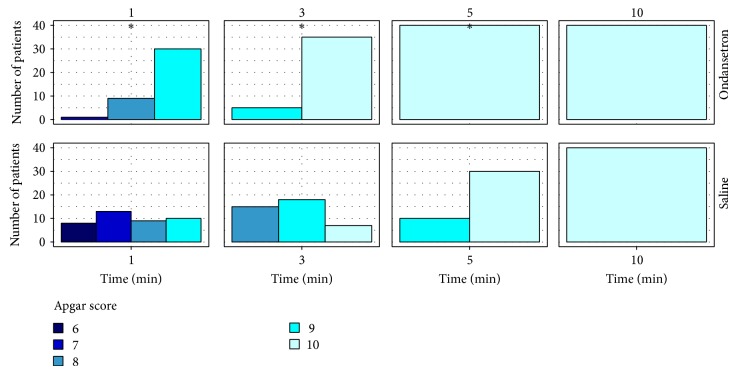
Apgar score of newborns during 1, 3, 5, and 10 minutes after birth. ∗ < 0.05.

Ondansetron was shown to attenuate arterial blood pressure drop due to spinal anesthesia in general surgery population in a study by Owczuk et al. [13] and in obstetrical population in a study by Sahoo et al. [14] (Figure 5). However, it was not shown to decrease this risk in obstetrical population in study of Ortiz-Gómez et al. [15]. A larger randomized controlled trial was performed here in order to further test the hypothesis that prophylactic ondansetron (5 mg) decreased the incidence of maternal hypotension following spinal anesthesia for elective caesarean section. Possible reasons include the specific population, sample size, study design, and anesthetic technique.

Our study compared ondansetron 5 mg (*n* = 40) with placebo (*n* = 40), Owczuk et al. [13] compared ondansetron 8 mg (*n* = 35) with placebo (*n* = 36), and Sahoo et al. [14] compared ondansetron 4 mg (*n* = 24) with placebo (*n* = 24) while Ortiz-Gómez et al.'s [15] study included three doses of ondansetron (2, 4, and 8 mg versus placebo).

Owczuk et al. [13] studied a general surgical population while Sahoo et al. [14] and Ortiz-Gómez et al. [15] studied obstetric patients undergoing caesarean delivery.

We consider the anesthetic technique the most important factor that might account for the difference between the studies.

Each of the three studies used a different dose of hyperbaric bupivacaine: we and Sahoo et al. [14] used 10 mg and Owczuk et al. [13] used 20 mg. In contrast, Ortiz-Gómez et al. [15] personalised each dose (mean dose of 9.7 ± 0.4 mg in the placebo group and 9.6 ± 0.3 mg in each of the ondansetron groups) to finally obtain doses which are slightly smaller than the 10 mg we used. This was interesting and may avoid over- or underdosing in women at the upper or lower extremes of height.

Differences in the design of the three studies should be noted. The first is the definition of hypotension. Owczuk et al. [13] did not supply a definition while Sahoo et al. [14] used a SBP < 90 mmHg or DBP < 60 mmHg. On the other hand, we (as well as Ortiz-Gómez et al. [15]) used the criteria outlined in the Cochrane review of hypotension in obstetrics [6].

Another difference is that Sahoo et al. administered intravenous fentanyl if the patient felt pain and tramadol or promethazine to treat adverse effects. These medications could modify blood pressure either directly or indirectly, possibly through a central mechanism.

Also, we routinely use intrathecal opioids to improve the quality of spinal anesthesia. This makes a direct comparison with the findings of Sahoo et al. [14] (but not with Ortiz-Gómez et al. [15] who used intrathecal fentanyl) difficult as the mechanism of action of ondansetron may be central and therefore affected by intrathecal opioids. Finally, we (as well as Ortiz-Gómez et al. [15]) did not use other supplemental analgesia and if required, those women were to be eliminated from the study.

Neither we nor Sahoo et al. [14] have reported their oxytocin protocol after umbilical cord clamping. This is important to explain results because of side effects which could affect maternal haemodynamics. Only Ortiz-Gómez et al. [15] have detailed oxytocin injection. They used low doses of (1 U), followed by an infusion of 2.5 U/h. Ours differ largely from this protocol. In fact we use bolus of 5 U in 5 minutes followed by an infusion of 2.5 U/h.

In conclusion, our results and those from Ortiz-Gómez et al., although methodologically close, differ because of different hypotension's management (type of loading fluids, type of vasopressor, and oxytocin protocol). This is sometimes due to economic constraints. For example, the unique vasopressor we have in our country is ephedrine (does not reflect current best practice) known to be associated with fetal acidosis [16, 17].

The strength of our study lies in two subjects. The first is “minimally invasive” hemodynamic parameter monitoring to try to explain the mechanism of action of ondansetron. We know that the use of the MOSTCARE device (which is not yet validated in obstetric parturients) is not excessive (compared to transesophageal echocardiography) for this indication and it took a long time for the local ethics committee to give its agreement for this study. Echocardiography would have been an alternative but unavailability led us to choose MOSTCARE.

It seems that ondansetron may act at cardiac level (enhancing contractility and efficiency) and also at vascular level (stable SVR) via vascular and/or medullar specific receptors. Studies including more invasive haemodynamic technique (i.e., Swan-Ganz catheter) could be helpful.

The second is the neonatal parameters, and we showed that ondansetron can be helpful to improve metabolic and vital parameters of newborns, in any case, under our skies.

## 6. Conclusion

Our study showed that prophylactic ondansetron had a significant effect on the incidence of hypotension in healthy parturients undergoing spinal anaesthesia with bupivacaine and sufentanil for elective caesarean delivery.

## Figures and Tables

**Table 1:** Demographic characteristics.

	Group O (*n* = 40)	Group S *n* = 40	*P*
Age (years)	33 ± 4	33 ± 4	*0.883 *
Weight (kg)	79 ± 11	77 ± 10	*0.447 *
HR (bpm)	90 ± 13	92 ± 11	*0.642 *
SAP (mm Hg)	124 ± 10	126 ± 11	*0.456 *
DAP (mm Hg)	80 ± 10	80 ± 11	*0.984 *
MAP (mm Hg)	95 ± 9	94 ± 10	*0.417 *
Weight of newborns (gr)	3443 ± 650	3372 ± 431	*0.567 *

HR: heart rate, SAP: systolic arterial pressure, DAP: diastolic arterial pressure, and MAP: mean arterial pressure.
